# Mechanisms and treatments of lymphedema

**DOI:** 10.3389/fimmu.2026.1827439

**Published:** 2026-04-23

**Authors:** He Ren, Yanhong Gong, Shuting Zhang, Jing Liu, Jiahe Shen, Oksana Gizinger, Tamara Aripova, Ju Liu

**Affiliations:** 1 Medical Research Center, Shandong Provincial Qianfoshan Hospital, Shandong University, Jinan, Shandong, China; 2 Laboratory of Translational Medicine in Microvascular Regulation, Medical Research Center, The First Affiliated Hospital of Shandong First Medical University & Shandong Provincial Qianfoshan Hospital, Jinan, Shandong, China; 3 School of Traditional Chinese Medicine, Shandong University of Traditional Chinese Medicine, Jinan, Shandong, China; 4 Department of Stomatology, The First Affiliated Hospital of Shandong First Medical University & Shandong Provincial Qianfoshan Hospital, Jinan, Shandong, China; 5 Shandong Provincial Key Medical and Health Laboratory of Translational Medicine in Microvascular Aging, Jinan, Shandong, China; 6 Laboratory for Future Industry in Gene Editing in Vascular Endothelial Cells of Universities of Shandong Province, Jinan, Shandong, China; 7 Department of Immunology and Allergology, Medical Institute, Peoples’ Friendship University of Russia (RUDN University), Moscow, Russia; 8 Institute of Immunology and Human Genomics, Academy of Sciences of Uzbekistan, Tashkent, Uzbekistan

**Keywords:** CD4^+^T cells, lymphangiogenesis, lymphatic endothelial cells, lymphedema, pathophysiology, therapy

## Abstract

Lymphedema refers to the interstitial edema caused by obstruction of lymph drainage. The clinical symptoms of lymphedema include local swelling and pain, thickened and rough skin, limited walking and repeated infection. Genetic mutation, fat deposition, aging and lymphatic filariasis may contribute to the pathogenesis of lymphedema. CD4^+^T cells also promote the development of lymphedema. Cell junctions between adjacent lymphatic endothelial cells (LECs) are crucial to maintain the integrity of lymphatic vessels, and damage of these junctions increases the vessel permeability and induces lymphedema. VEGF-C-VEGFR3 signaling is a key regulator of lymphangiogenesis, whereas inflammatory mediators modulate lymphatic repair in a context-dependent manner. Current management includes conservative therapy and selected surgical approaches, while lymphangiogenic, pharmacologic, and cell-based treatments remain largely investigational. In this review, we summarize pathogenic factors associated with lymphedema, mechanisms regulating lymphatic endothelial permeability and lymphangiogenesis, and emerging therapeutic strategies.

## 1 Introduction

Lymphedema is a chronic and progressive disorder of the lymphatic system characterized by persistent inflammation, adipose deposition, and tissue fibrosis (1). It can be divided into primary and secondary forms based on the underlying etiology. Primary lymphedema is mostly related to the injury caused by gene mutation (2), whereas secondary lymphedema is commonly associated with trauma, infection,or therapeutic intervention (2, 3). Persistent swelling and fibrosis markedly impair patient quality of life (4). Current treatment options remain limited and include conservative, surgical, and emerging regenerative approaches. This review summarizes current mechanisms and treatment strategies in lymphedema.

## 2 Lymphatic network

**Figure 1:**
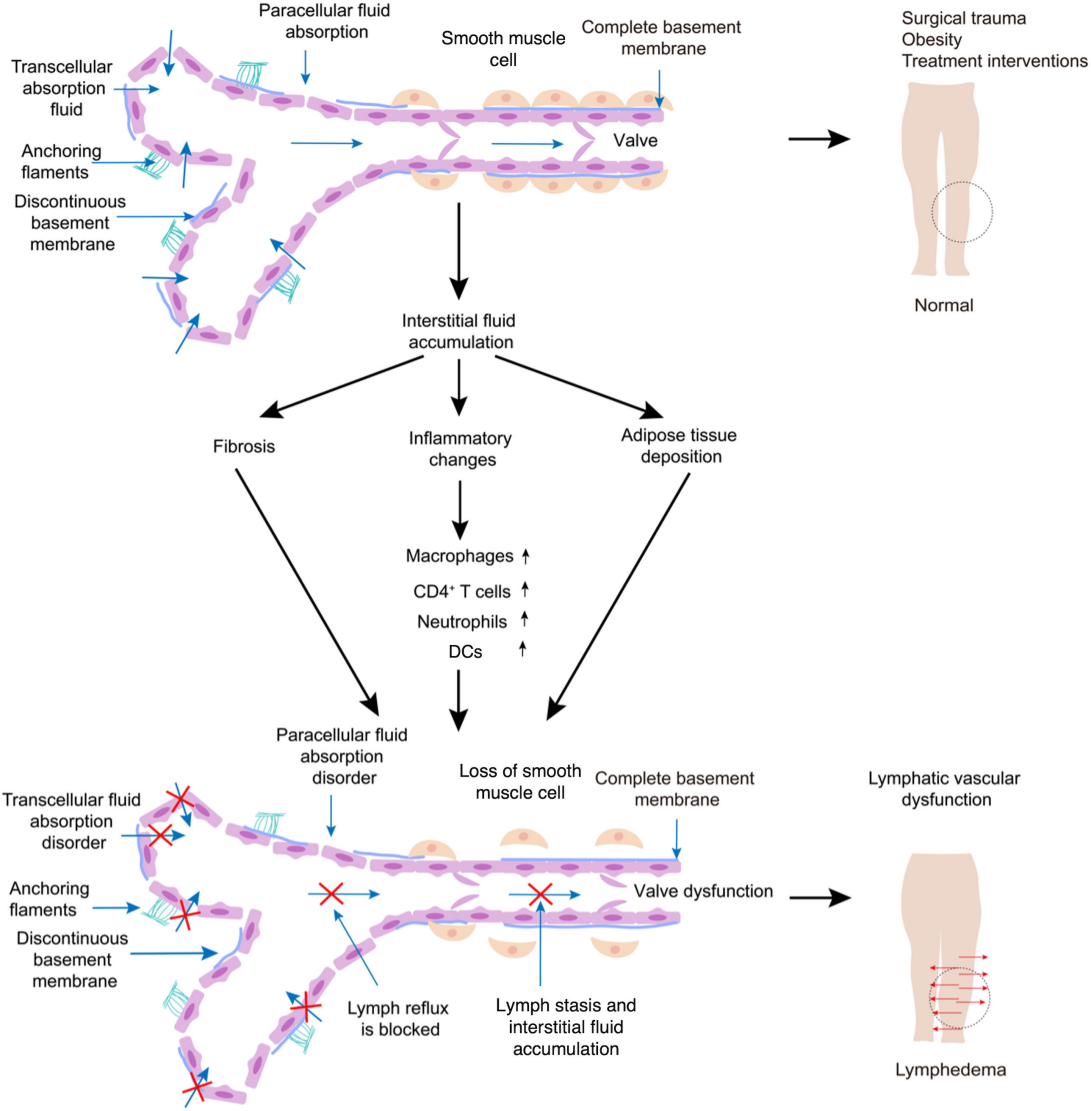
Pathophysiology of secondary lymphedema. The initial lymphatic capillaries are composed of monolayer lymphatic endothelial cells (LECs) that are connected with each other in the form of buttons. LECs are anchored to the cell matrix through anchored filaments. These specific structures may conduce to the entry of interstitial fluid and cells into the lymphatic capillaries. Many lymphatic capillaries aggregate to form collecting lymphatic vessels. There are valves on the lymphatic vessel wall, and smooth muscle is connected to the lymphatic vessel outside the lymphatic vessel wall. Lymphatic valves allow only unidirectional lymph flow. Due to surgery, obesity or treatment injury, lymphatic reflux is damaged, lymphatic stasis, tissue colloid osmotic pressure increases, and tissue fluid accumulates outside the cells, causing lymphedema. The hallmark pathological feature of secondary lymphedema is retention of interstitial fluid. Persistent fluid retention promotes chronic inflammatory responses, including infiltration of CD4^+^ T cells, macrophages, neutrophils and DCs, as well as fat accumulation and tissue fibrosis. Prolonged lymphatic injury may lead to lymphatic dysfunction, loss of smooth muscle and valve injury.

The lymphatic system is composed of lymphatic vessels, lymphoid organs and tissues. They work synergistically to absorb and transport the fluid against gravitational and pressure gradients (5). The lymphatic capillaries are composed of a single layer of oak-leaf-shaped endothelial cells with a discontinuous and unclear basal membrane (6). There are discontinuous button-like junctions between LECs, endowing the lymphatic capillaries with high permeability (6). The lymphatic capillaries allow the entry of interstitial fluid, macromolecules, lipids and immune cells (5). When the tissue pressure changes, the LECs adhere to the extracellular matrix by anchoring filaments, ensuring the normal entry of tissue fluids (7–9). The absorbed fluid, known as lymph, flows towards collecting lymphatics (10). The collecting lymphatics have continuous zipper-like junctions and lymphatic muscle cells (6, 10), which provide the structural basis for the propulsion of lymph (**Figure 1**) (6, 11). Lymph is transported through afferent collecting lymphatic to lymph nodes (6). Efferent collecting lymphatics then transport the lymph to the thoracic duct (12). The lymph then drains into the left and right subclavian veins to complete lymph circle (13, 14). The lymphatic system maintains fluid homeostasis, lipid absorption, and immune-cell trafficking (15–17). Under pathological conditions, failure of this transport network leads to lymphatic stasis and lymphedema.

## 3 Pathophysiology of lymphedema

### 3.1 Primary lymphedema

#### 3.1.1 VEGFR3 signaling defects in Milroy disease

Primary lymphedema is mostly related to the injury caused by gene mutation (2). Milroy’s disease is an autosomal hereditary lymphedema (18, 19). Patients usually exhibit lymphedema at birth with the swelling of the lower limbs (20). This disease is caused by a mutation of vascular endothelial growth factor receptor-3 (*VEGFR3*) (18, 19), which has been mapped to the telomeric part of chromosome 5q34-q35 (19). VEGFR3 is a receptor tyrosine kinase specific for lymphatic vessels (19). Studies have found that VEGFR3 might bind to surface ligands of LEC to regulate lymphatic function (20). However, protein products from mutated *VEGFR3* have no tyrosine kinase activity and are unable to bind to EC surface ligands (21). With the increase of VEGFR3 mutants, the proliferation, differentiation and migration of LECs are inhibited, resulting in lymphatic dysfunction and lymphatic reflux blockade (22). Consequently, interstitial lymph accumulation drives inflammation, fibrosis, and adipose deposition, which exacerbates edema and culminates in Milroy’s disease (20, 23).

#### 3.1.2 FOXC2 dysfunction in lymphedema-distichiasis

Lymphedema-distichiasis (LD) is another autosomal dominant disorder. LD is typically characterized by lower limb lymphedema and valve abnormalities (24–26). LD is linked to dominant mutations of Forkhead box protein C2 (*FOXC2*) gene (27–29). *FOXC2* mainly controls the mechanoresponses of LECs. Abnormal mechanotransduction is the primary cause of the defects observed in patients with LD (24). In animal experiments, 25% of *Foxc2*^+/–^ mice were reported to develop defects in former valves of lymphatic vessels (24, 30). The absence of FOXC2 can disrupt the integrity of lymphatic intercellular connections, resulting in increased endothelial permeability (24), lymphatic leakage, and ultimately lymphedema (24).

#### 3.1.3 Additional genetic causes of primary lymphedema

Many other gene mutations are associated with lymphedema. For example, variation of SRY-related HMG-box 18(*SOX18*) gene causes hypotrichosis-lymphedema-telangiectasia syndrome (31). GATA-binding protein 2 (*GATA2*) mutation leads to Emberger syndrome (32). In addition, mutation of genes, including hepatocyte growth factor (*HGF*) (33), kinesin family member 11(*KIF11*) (34), protein tyrosine phosphatase, nonreceptor type 11(*PTPN11*) (35), *KRAS* (35), son of sevenless 1 (*SOS1*) (35), *RAF1* (35), NF-κβ essential modulator (*NEMO*) (36), also known as p120 RasGAP(*RASA1*) (37–39), *HRAS* (14, 40, 41), ankyrin repeat and PH domain 3(*ARAP3*) (42), RAR-related Orphan Receptor C(*RORC*) (43), and semaphorin 3A (*SEMA3A*) (44) have been reported to cause lymphedema.

### 3.2 Secondary lymphedema

#### 3.2.1 Tissue injury and lymphatic stasis

Secondary lymphedema is more common than primary lymphedema. Many factors, such as surgical trauma, recurrent infection, obesity, parasites, malignancies and lymphatic injuries caused by treatment intervention (45–49), can lead to lymphatic dysfunction, including enlargement of lymphatic vessels, absence of smooth muscle and damage of valves (50, 51). Interstitial fluid accumulation is an early consequence of lymphatic injury and promotes subsequent inflammation, fibrosis, and adipose deposition (2).Persistent lymphatic damage ultimately impairs interstitial fluid transport and leads to clinically overt lymphedema (3).

#### 3.2.2 Immune activation and T-cell-driven progression

CD4^+^T cells might mediate the above pathological consequence directly or indirectly (52). Ly et al. created a mouse model of relative CD4^+^T cells deficiency by transplanting bone marrow progenitor cells derived from CD4 knockout (CD4KO) mice into wild-type (WT) mice followed by lethal whole-body irradiation. They found that small numbers of CD4^+^T cells induce the development of lymphedema (53). Avraham et al. found that in mouse models of lymphedema, nude and CD4KO mice showed reduced tissue fibrosis, significantly improved lymphatic function and reduced tail lymphedema compared with WT mice (54). Another study demonstrated that CD4KO mice that underwent lymphatic injury without subsequent adoptive transplantation had the mildest degree of tail lymphedema. Thus, the depletion of CD4^+^T cells might contribute to the restoration of lymphatic function and normal lymphatic return, thereby preventing the development of lymphedema (54). Campbell et al. performed T-cell receptor(TCR) sequencing on paired normal and lymphedematous skin biopsies and found increased CD4^+^T cell clonality (oligoclonal expansion) in lymphedematous tissue; expanded clones were largely patient-specific, consistent with local antigen-driven activation (55).

Beyond CD4^+^T cells, other immune populations also participate in lymphedema progression. Flow-cytometric analyses in mouse models showed significant increases in neutrophils, macrophages, and dendritic cells in lymphedematous tissues, whereas after axillary lymph node dissection the early inflammatory response was more prominently associated with T-helper-cell expansion, suggesting that adaptive immune activation may precede macrophage and dendritic-cell accumulation (56). García Nores et al. further showed that naïve CD4^+^T cells are activated in skin-draining lymph nodes after interaction with antigen-presenting cells and then migrate to the skin, where they promote fibrosis, inflammation, and lymphatic dysfunction (52).In parallel, single-cell transcriptomic analysis of cancer-associated lymphedema identified depletion of LYVE1+ anti-inflammatory macrophages and enrichment of pro-inflammatory TREM1+ macrophages; pharmacologic Trem1 blockade alleviated edema and fibrosis in a mouse tail model (57). Together, these findings indicate that dendritic cells are mainly involved in CD4^+^T-cell activation, macrophages contribute to inflammatory amplification and tissue remodeling, whereas evidence for neutrophils is currently more descriptive and remains less mechanistically defined. Overall, CD4^+^T cells remain the best-characterized effector population in lymphedema pathogenesis, although much of the mechanistic evidence is still preclinical and human data remain limited and largely associative (52–54).

#### 3.2.3 Stromal remodeling and fibrotic signaling

Recent studies have further clarified the cellular and molecular basis of lymphedema. Single-cell RNA sequencing of subcutaneous adipose tissue from cancer-associated lymphedema identified an expanded PRG4+/CLEC3B+ adipose-derived stromal-cell subset associated with adipose fibrosis (57). CLEC3B was upregulated in patient-derived ASCs, and its knockdown attenuated extracellular-matrix programs and reduced fibrogenic readouts together with decreased SMAD2/3 signaling, supporting a functional role for CLEC3B in stromal activation. In parallel, TREM1 was preferentially expressed by pro-inflammatory macrophages, and pharmacologic Trem1 blockade alleviated lymphedema in a mouse tail model (57). These findings suggest that CLEC3B-driven stromal activation and TREM1-mediated macrophage amplification converge on SMAD-dependent profibrotic signaling. In breast cancer-related lymphedema, TGF-β1 and downstream pathways are activated in diseased tissues (58), and TGF-β1 neutralization reduces extracellular matrix deposition and immune-cell infiltration (59). Consistently, enhanced TGF-β1/Smad2/3 signaling has been observed in both experimental and clinical lymphedema, while treatment with the ALK5 inhibitor EW-7197 reduced tail swelling, decreased α-SMA expression, and enhanced lymphangiogenesis and interstitial flow in a mouse model of acquired lymphedema (60).

#### 3.2.4 Adipose-lymphatic crosstalk and obesity

Adipose deposition is a characteristic feature of chronic lymphedema and becomes clinically apparent with disease progression (61). Recent studies have found a bi-directional crosstalk between lymphatic vessels and adipose tissue (10). Lymphatic vessel damage results in subcutaneous adipose tissue deposition, and ultimately obesity (62). Prospero homeobox-1 (Prox-1) is involved in the induction of lymphoendothelial phenotype and lymphatic vessel formation. Embryonic Prox-1 null mice develop lymphatic dysplasia and display edema (63). The few surviving pups that reach adulthood developed lymphatic leakage, fat accumulation and obesity (63). On the other hand, extreme obesity might damage lymphatic vessels, leading to lower extremity lymphedema (64). In a diet-induced obese mouse model, impaired lymphatic transport was observed (65). In addition, postoperative lymphedema is also associated with obesity. In a Meta-analysis, the body mass index of female breast cancer patients with lymphedema was significantly higher than that of normal controls (66). In brief, this bidirectional regulatory effect between obesity and lymphedema might lead to lymphatic dysfunction and even aggravate the development of lymphedema.

## 4 Malfunctions of LECs in lymphedema

### 4.1 Role of LEC junctional molecules in lymphedema

In collecting lymphatic vessels, LECs form continuous zipper-like junctions together with bileaflet valves, which ensure directional lymph flow and limit fluid exchange with surrounding tissues. LECs contacts are formed by junctional adhesion molecules, including tight and adherens junctions. They maintain the integrity, stability, and molecular permeability of the intercellular contact between LECs (67). These tight and adherens junctions are composed of vascular endothelial cadherin (VE-cadherin) and tight junction related proteins, including occludin, claudin-5, zonula occludens-1, junctional adhesion molecule-A (JAM-A), and endothelial cell-selective adhesion molecule (ESAM) (6).Mechanical cues including fluid shear stress are sensed by junction-associated components, notably PECAM and VE-cadherin, together with VEGFR2/VEGFR3, and can activate PI3K/Akt signaling to regulate cytoskeletal organization and endothelial barrier function (68). In lymphedema, leaky lymphatics have been associated with discontinuous expression of junctional proteins, particularly VE-cadherin and claudin-5, and are often accompanied by non-functioning lymphatic valves (61).

VE-cadherin is the main component of adhesive junction and is essential for the development of lymphatic vessels. It is required for the development of lymphatic venous valves in embryos (69). Cdh5 is the coding gene for VE-cadherin. *Cdh5^flox/flox^* and *Prox1CreER^T2^* strains were interbred to generate *Cdh5^flox/flox^* controls and *Prox1CreER^T2^*; *Cdh5^flox/flox^* (*VE-cadherin^LEC-Ko^*) mouse models. *VE-cadherin^LEC-Ko^* embryos had lymphatic flap deletion in E14.5 and E16.5, as well as severe edema (69). The mesenteric lymphatics of adult mice are more susceptible to VE-cadherin loss and cause mesenteric lymphatic obstruction.

Coxsackie virus and adenovirus receptor (CAR) is one of the tight junction molecules. It participates in lymphatic vessel formation together with junction adhesion molecule (JAM) and ESAM in adult tissues (70). Recent studies suggest that lymphatic endothelial cells of CAR-deficient mouse embryos have incomplete tight junctions (71). Another study showed that siRNA-mediated knockdown of CAR expression in cultured LECs led to a reduction in intercellular adhesion (70). Downmodulation of CAR might affect cellular connections between LECs, leading to abnormal lymphoid function, increased permeability, and accumulation of tissue fluid, ultimately leading to lymphedema (70, 72).

### 4.2 Lymphangiogenesis and lymphedema

Lymphangiogenesis is the formation of lymphatic vessels from pre-existing lymphatic vessels, including proliferation, migration and lumen formation of LECs. Physiologically, lymphangiogenesis primarily occurs during embryonic development and wound healing. Lymphangiogenesis is uncommon in healthy adults. Pathologically, impaired lymphangiogenesis further inhibits the proliferation, migration and differentiation of LECs, exacerbates persistent fluid accumulation, leading to lymphedema (73). The discovery of growth factors and receptors of lymphatic vessels reveals the regulatory mechanism of lymphangiogenesis.

VEGFR3 is mainly expressed in LECs. As a receptor tyrosine kinase, VEGFR3 signaling promotes lymphangiogenesis. The ligand VEGFC specifically binds to VEGFR3 and activates downstream signaling pathways to promote phosphorylation of phosphoinositol 3-kinase (PI3K)/Akt and MAPK, thereby inducing proliferation, migration and survival of LECs and promoting lymphangiogenesis (74). β1 integrin also interacts with VEGFR3 to enhance VEGFR3 signaling, mechanically inducing LEC proliferation and promote lymphangiogenesis (75).

Inactivation of VEGFR3 tyrosine kinase is responsible for most hereditary lymphedema (19). Recently, mutations in the VEGFR3 gene have been found to cause Hennekam syndrome (76–79). Hennekam syndrome typically presents with lymphedema (80). In addition, Hennekam syndrome also associates with mutations in collagen and calcium binding EGF domains 1 (*CCBE1*) and ADAM metallopeptidase with thrombospondin type 1 motif 3 (*ADAMTS3*) (81, 82). The mutated *CCBE1* and *ADAMTS3* might promote proteolysis of VEGFC and interrupt the VEGFR3 signaling pathway of lymphangiogenesis (76–79).

Inflammatory mediators are integral to secondary lymphedema. Although inflammation is generally associated with increased vascular and lymphatic permeability (1), selected inflammatory signals can modulate lymphangiogenesis in a dose-dependent manner.LTB4 is an inflammatory lipid mediator. LTB4 at high concentrations in the range from 200 to 400 nM inhibit VEGFR3 phosphorylation and lymphangiogenesis, exacerbating lymphedema. However, lower LTB4 concentrations ranging from 1 to 10 nM were prolymphangiogenic (83).

IL-8 can also promote lymphangiogenesis through promoting the proliferation, migration and survival of LECs, and ultimately improve lymphedema (84). In addition, type 1 cytokines including interleukin 1(IL-1), interleukin 12 (IL-12), interleukin 18 (IL-18), and tumour necrosis factor alpha (TNF-α) might promote lymphangiogenesis and reduce lymphedema (85).

Other signaling factors of lymphogenesis, including fibroblast growth factor 2 (FGF2), FGF receptor 1 (FGFR1), sphingosine-1-phosphate (S1P), S1P receptor I (S1PRI), and bone morphogenetic protein 9 (BMP9), and activin receptor-like kinase 1 (ALK1) also regulate the development of lymphedema by regulating lymphangiogenesis (78).

## 5 Therapies for lymphedema

Due to the disfiguring and disabling nature, lymphedema affects the quality of life and mental health of patients (86). Therefore, early prevention and treatment of lymphedema is particularly important. Although early identification is critical, patients frequently report delays in diagnosis and treatment, underscoring deficiencies in routine care and the need for standardized management pathways (87). Current management includes conservative therapy, selected surgical interventions, and emerging investigational approaches. Conservative treatment remains the clinical foundation, whereas physiologic or reductive surgery is considered in selected patients according to disease stage and residual lymphatic function. By contrast, pharmacologic, lymphangiogenic, and cell-based strategies remain largely investigational, with most evidence derived from preclinical studies or early-phase clinical trials.

### 5.1 Nonsurgical therapy treatment

In conservative treatment, lymphatic drainage and massage might alleviate symptoms. Combined decongestant therapy(CDT) is the main method of physical therapy for lymphedema (88). CDT is a two-stage treatment plan jointly formulated by lymphologists and physiotherapists. The first stage mainly includes skin care, manual lymphatic drainage (MLD), the use of multi-layer compression bandages and exercise. The second stage, which is mainly regarded as the maintenance stage, includes skin care, self MLD, the use of pressure socks and exercise (86). Other physical treatments, including lymphatic drainage, massage, weight loss and exercise, can also relieve the symptoms of edema. Drug therapy is another treatment for lymphedema. Gardenier et al. studied the local effect of tacrolimus in a mice lymphedema model (89). Results have shown that tacrolimus reduces tissue fibrosis, decreases CD4+ cell infiltration, increases lymphatic reflux and increases lymphatic function (90). In addition, high physiological doses of vascular endothelial growth factor-C (VEGF-C) administered locally to lymphedema model animals significantly increased lymphangiogenesis and reduced swelling (91). VEGF-C-based therapy has also been evaluated in clinical studies. Lymfactin^®^ is an adenoviral vector encoding human VEGF-C and has been used together with vascularized lymph node transfer in breast cancer-related lymphedema. Long-term follow-up showed no serious adverse events and improved quality-of-life scores (92). Apelin (APLN), a vascular regulatory peptide acting through the APJ receptor (93), has been evaluated as an adjunct to VEGF-C therapy. Mechanistically, APLN promotes CCBE1-dependent VEGF-C maturation and activates AKT/ERK and eNOS signaling, enhancing lymphatic endothelial migration and collecting-vessel pumping (94). In a mouse model of secondary lymphedema, APLN-VEGF-C mRNA delivery reduced limb swelling and improved dermal backflow (94). However, these pharmacologic and pro-lymphangiogenic strategies should still be interpreted cautiously, as most data remain preclinical and direct comparison across studies is limited by differences in model systems, dosing, and outcome assessment.

### 5.2 Surgical therapy

Surgical management may be considered in patients with persistent symptoms despite optimized conservative therapy. Current procedures can be broadly divided into physiologic reconstruction, reductive surgery, and preventive microsurgical approaches (95).

Physiologic procedures, primarily lymphaticovenous anastomosis(LVA) and vascularized lymph node transfer (VLNT), aim to restore or augment lymphatic drainage, and are generally more applicable to patients with residual functional lymphatics or potentially reversible disease (95, 96). Indocyanine green lymphography is commonly used for operative planning. In early-stage breast cancer-related lymphedema, LVA improved patient-reported physical and mental function at 6 months, although limb volume reduction was not significant at that time point (97). This stage dependence is clinically important, because patients with residual functional lymphatics and less advanced fibroadipose remodeling are more likely to benefit from physiologic reconstruction, whereas extensive fibrosis may limit benefit in later-stage disease.

In addition to clinical improvement, mechanistic studies suggest that LVA may reduce local inflammation and fibrosis, with decreases in CD4+ cell infiltration, collagen type I deposition, and TGF-β1 expression (98), while postoperative immune profiling has also shown reduced inflammatory signaling and increased T-cell receptor diversity (99).

VLNT represents an alternative physiologic strategy when native lymphatic channels are severely compromised. Meta-analytic evidence suggests improvements in limb volume, quality of life, and infection burden, although the available studies remain heterogeneous and largely nonrandomized (100). Experimental and translational observations further suggest that lymph node transfer may exert anti-inflammatory and pro-lymphangiogenic effects, including IL-10- and VEGF-C-related changes, but these findings remain preliminary (101, 102).

Preventive approaches, including axillary reverse mapping (ARM), the lymphatic microsurgical preventive healing approach(LYMPHA), and immediate lymphatic reconstruction (ILR), are intended to reduce the risk of cancer-related lymphedema at the time of oncologic surgery (103). By contrast, reductive procedures such as suction-assisted protein lipectomy or excisional debulking are mainly used for later-stage disease with fibroadipose deposition; these operations reduce limb volume but do not restore lymphatic function (95). Overall, current evidence supports a stage-adapted surgical strategy rather than a uniform operative approach. Physiologic reconstruction benefits selected patients with salvageable lymphatic function, whereas reductive surgery mainly addresses advanced fibroadipose disease. Preventive microsurgery is appropriate for high-risk patients undergoing cancer treatment. Importantly, much of the surgical literature remains observational and center-dependent, highlighting the need for standardized staging, prospective comparisons, and longer follow-up.

### 5.3 Cell therapy

Cell-based therapy has attracted interest as a potential regenerative strategy for lymphedema because transplanted cells may support lymphangiogenesis and tissue repair. Mesenchymal stem cells, particularly adipose-derived cell populations, have been studied most extensively and have shown pro-lymphangiogenic and reparative effects in experimental settings (104–106). However, the current clinical evidence remains limited to small early-phase studies with heterogeneous cell products, delivery protocols, and outcome measures. Therefore, cell-based therapy should currently be regarded as an investigational rather than an established treatment for lymphedema. Representative clinical studies, together with their methodological characteristics and key limitations, are summarized in **Table 1**.

**Table 1:** Cell-based therapy of human lymphedema.

Edema type	Study design	Cell type	Injection method (location/depth)	Results	Key limitations
Arm breast cancer associated lymphoedema (106)	Case report	Freshly isolated adipose-derived stromal cells	Axillary region/Subcutaneous	1. Symptoms of arm heaviness and tension were alleviated. 2. Perimeter of arm↓ 3. Armvolume↓ 4. No postoperative complications or adverse events.	Single case; no postoperative complications or adverse events reported.
Post-mastectomy upper-extremity lymphedema (107)	Prospective randomized pilot study	Autologous bone marrow-derived mononuclear/CD34+ cells	Around the axillary/Intramuscular injection	1. Armvolume↓ 2. Pain and sensitivity in the arm were reduced.	Small pilot study; short follow-up; no lymphoscintigraphy was performed.
Breast cancer-related lymphedema (108)	Feasibility and safety study	Freshly isolated adipose-derived regenerative cells	Axillary/Subcutaneous	1. No serious adverse events. 2. Half of the patients reduced their use of conservative management. 3. Armvolume↓	Small single-arm pilot study.
Chronic lower-limb lymphedema (109)	Randomized controlled clinical trial	Bone Marrow-Derived Mononuclear Cells	Left lower limb/Subcutaneous	1. Circumference of limbs↓ 2. Pain in the limb was reduced. 3. Walking ability in patients ↑	Single-center study; limited long-term data.
Breast cancer associated lymphoedema (BCRL) (110)	Open-label phase I trial; 4-year follow-up of the previously reported cohort	Adipose-derived regenerative cells	Axillary region/Subcutaneous	1. Patients reported improved arm, shoulder and hand function. 2. Six of the 10 patients reduced the use of conservative treatment for lymphedema.	Small single-arm long-term follow-up; no serious adverse events; no locoregional recurrence reported.

## 6 Discussion

Lymphedema is a progressive disorder sustained by lymphatic injury, immune activation, endothelial dysfunction, and stromal remodeling. CD4^+^T cells remain the best-characterized immune component in experimental models, while growing evidence also supports contributions from macrophage-associated inflammatory amplification and profibrotic signaling. These processes converge on impaired lymphatic repair, fibrosis, and adipose deposition, thereby promoting disease progression.

From a clinical perspective, this mechanistic heterogeneity helps explain why treatment response is also variable. Conservative therapy remains the basis of management, whereas physiologic surgery appears most appropriate in selected patients with residual lymphatic function and less advanced fibroadipose remodeling. By contrast, reductive procedures primarily address later-stage volume burden rather than restoration of lymphatic physiology. Pharmacologic, lymphangiogenic, and cell-based strategies remain promising, but their current evidence base is still limited by heavy reliance on preclinical models, small early-phase clinical studies, and inconsistent endpoints. For this reason, mechanistic rationale alone should not be interpreted as established clinical efficacy.

Current limitations in the field include heterogeneous staging systems, inconsistent outcome measures, and limited mechanistic biomarkers, all of which restrict comparison across studies and hinder precision treatment selection. Future work should integrate standardized clinical staging with imaging- and biomarker-based stratification to better align patients with conservative, surgical, or investigational therapies.
